# Athens multifamily therapy project

**DOI:** 10.1192/j.eurpsy.2021.1332

**Published:** 2021-08-13

**Authors:** M. Selakovic

**Affiliations:** Psychiatric Department, GH Sismanoglio, Athens, Greece

**Keywords:** FEP, multifamily, systemic, psychotherapy

## Abstract

**Introduction:**

The Athens multifamily therapy project (A- MFTP) provides systemic multifamily group therapy to youths who experienced a first psychotic episode (FEP) and their families.

**Objectives:**

The participants were recruited from the ongoing longitudinal Early Psychosis Intervention Study –ELPIS, Athens FEP Project, which aims to investigate the involvement of genetic and environmental determinants on psychosis risk.

**Methods:**

A group of five families with a child who had experience FEP, attended two multifamily group sessions per month, in the time period from September 2017 to Jun 2018. Parents and offspring participated to the sessions, which were conducted by two co-therapists. Assessment of patients’ psychopathology was based on PANSS at baseline, end of therapy and 6-month follow-up. All participants fulfilled an instrument assessing family factors (SCORE-15) and the Reflective Functioning Questionnaire (RFQ) at the same three time points. Furthermore, participants were asked to give written opinions regarding the therapeutic process at the middle phase, the end of therapy and six months follow - up.

**Results:**

A qualitative analysis identified the emerging themes and patterns, focusing on the language and the meaning constitutes. Communication techniques, emotional processing and problem solving were the main learnings for the members of the group. They highlighted the impact of the group processes on family communication and individual understanding, while the development of a “new family” emerged from the group relationships.

**Conclusions:**

A- MFTP seems to be a promising service aiming to improve mental health and wellbeing of participants, to contrast chronicity and to contribute to early intervention services for psychoses in Greece.

